# Hemodynamic parameters after Delayed Cord Clamping (DCC) in term neonates: a prospective observational study

**DOI:** 10.1186/s12887-022-03303-4

**Published:** 2022-05-06

**Authors:** Bhvya Gupta, Rameshwor Yengkhom, Nishant Banait, Chinmay Chetan, Prince Pareek, Pradeep Suryawanshi

**Affiliations:** 1 Sparsh Superspeciality Hospital, Ambala city, Haryana India; 2 Jawaharlal Nehru Institute of Medical Sciences, Imphal, Manipur India; 3 All India Institute of Medical Sciences, Nagpur, Maharashtra India; 4 Himalayan Institute of Medical Sciences, Dehradun, Uttarakhand India; 5 Department of Neonatology, Bharati Vidyapeeth University Medical College, Dhankawadi Pune, Maharashtra 411043 India

**Keywords:** Delayed cord clamping (DCC), Transitional circulation, Functional echocardiography (FnEcho)

## Abstract

**Background:**

Delayed cord clamping (DCC) is practiced worldwide, as standard care in both term and preterm babies. Our aim was to determine the hemodynamic effects of DCC on transitional circulation.

**Material and methods:**

This prospective observational study was carried out in a tertiary care hospital, at Pune, India, from May 2018 to October 2019.Term neonates born during the study period were included. The primary outcome variables of the study were right ventricular output (RVO), left ventricular output (LVO), superior vena cava (SVC) flow (ml/kg/min) and heart rate(HR) at 12 ± 6 and 48 ± 6 h of life measured by point of care functional echocardiography. Inter-observer and intra-observer variability was calculated for these parameters.

**Results:**

Out of a total of 2744 deliveries during the study period, 620 babies were included. Mean gestational age of the enrolled babies was 38.96 ± 1.08 weeks and mean birth weight was 2.9 ± 0.39 kg. Mean heart rate of babies recorded at 12 ± 6 h of life was 127 beats per minute (bpm) whereas it was 128 bpm at 48 ± 6 h of life. RVO {mean (SD)} was 209.55(44.89) and 205.85(46.35) ml/kg/min, LVO {mean (SD)} was 133.68(31.15) and 134.78(29.84) ml/kg/min whereas SVC flow {mean (SD)} was 106.85(26.21) and 109.29(25.11) ml/kg/min at 12 ± 6 and 48 ± 6 h of life respectively. There was good intra-observer agreement in all the variables.

SGA babies had a significantly higher heart rate at 12 ± 6 h of life as compared to AGA babies, although this difference in heart rate could not be appreciated at 48 ± 6 h of life. However SGA babies had a higher LVO, RVO and SVC flow than AGA babies at both the time points of observation.

**Conclusion:**

After DCC there is less fluctuation in the hemodynamic parameters (heart rate, cardiac output) at the two time points of observation.. As compared to AGA babies, SGA babies had a significantly higher baseline heart rate, LVO, RVO and SVC flow. LVO of SGA babies after delayed cord clamping is found to be significantly lower than LVO seen in other studies, favoring the cardio-stabilizing effect of DCC.

**Brief rationale:**

This is the first study with a large sample size evaluating the hemodynamic effects of DCC in term neonates by functional echocardiography. The normative data of heart rate and cardiac output of term, stable babies with small for gestational age(SGA) as a special subgroup undergoing DCC requires further evaluation.

## Background

Clamping and cutting of umbilical cord at birth is thought to be an ancient and prevalent intervention amongst humans. In spite of this, the optimal timing of umbilical cord clamping has been a debatable issue since ages [1]. Early cord clamping (defined as umbilical cord clamping at less than 60 s after birth) is believed to a reduce risk of postpartum hemorrhage [2]. Late cord clamping, synonymous with delayed clamping is a physiological approach which involves clamping of the umbilical cord when cord pulsations have ceased. However, there is a wide variation in definitions of early and late cord clamping [3]. The process of placental transfusion by DCC, can provide the neonate with 30% extra blood volume and around 60% additional red blood cells [3]. Advantages associated with DCC include higher hemoglobin levels [3], better cardiopulmonary adaptive status [4], supplementary iron stores and lesser degree of anemia in neonates up to six months of age [5]. The American College of Obstetricians and Gynecologist recommend a delay of at least one minute in umbilical cord clamping for vigorous term and preterm neonates [6].

There is insufficient understanding of the hemodynamic changes after placental transfusion as a result of DCC. All the studies found in the literature search regarding cardiac output measurement till date have tried to estimate normal cardiac output in term and preterm neonates during the transitional circulation. However, the timing of cord clamping and the effect of placental transfusion, which may have a significant effect on cardiac output and may lead to an alteration of the measured normative value of cardiac output has not been taken into consideration in any of these studies. Rather the lack of acknowledgement of timing of cord clamping and its influence on cardiac output has been mentioned as a limitation of many of the reviewed studies. Moreover the innate differences in the hemodynamic adaptability during transitional circulation especially with placental transfusion in term AGA vs SGA infants has not been studied so far. So,this study was designed with an aim of measuring the hemodynamic parameters by functional echocardiographic (FnEcho), after DCC in term neonates within the first 48 h of life.

## Methods

A prospective observational study carried out in a tertiary care referral hospital of Pune, Maharashtra, India. The enrolment period was May 2018 to October 2019. In this study, term neonates born intramurally during the study period after 37 completed weeks of gestation, vigorous after birth and not requiring NICU care were included after informed consent by parents.

Babies were excluded based on the following criteria:

- Preterm neonates (< 37 weeks) and Post term neonates (> 42 weeks)
- Neonates with perinatal depression requiring resuscitation at birth
- Neonates requiring NICU admissions in first 48 h of life
- Large for gestational age neonates (LGA)
- Multiple gestation
- Neonates with major congenital anomalies and genetic syndrome

### Study procedure

The study was commenced after approval by institutional ethics committee Bharati Vidyapeeth Deemed University Vide letter number BVDUMC/IEC/66. After written, informed consent, details of demographic characteristics like name, gender, date of birth, time of birth, birth weight, and gestational age, maternal variables (parity, pregnancy induced hypertension, gestational diabetes mellitus, thyroid disease, anemia, systemic illness) were collected in the clinical record sheet. All methods were carried out in accordance with relevant guidelines and regulations. Gestational age was calculated from 1st trimester USG/ LMP (Last Menstrual Period)/ New Ballard scoring system in the same order of preference as per availability. Significant antenatal and birth history (gestation, mode of delivery, birth weight, time of birth, APGAR score, time of cord clamping, resuscitation details if required) were recorded. Cord was clamped universally after 1 min, which is a routine protocol in the institute being practiced as a standard of care. The protocol of delayed cord clamping for all neonates was followed at birth (except for neonates requiring resuscitation and immediate cord clamping at birth).

### How DCC was achieved

Prior to enrollment of babies for the study, the routine practice of DCC in the institution was emphasized and endorsed by weekly lectures. Posters of routine policy were put up in the labor room/OT of the institution. At the time of delivery a stopwatch with alarm after 1 min was used to indicate the time of clamping of cord. In normal vaginal delivery, baby after delivery was put on mother’s abdomen for skin to skin contact and routine neonatal care was given there only. For caesarean delivery, baby was received in a pre-warmed linen and held wrapped by one member of the obstetric team for a duration of 1 min till the cord was clamped.

Birth weight was measured on a digital electronic scale (Kidlee®) reading to nearest 2 g. Echocardiographic measurements were performed by a single neonatologist trained in functional echocardiography with an experience of 10 years, using a Siemens ultrasound machine (Acuson X 300, Siemens Medical Solutions, Inc. USA) with probe (4–8 MHz transducer). First measurement was taken at 12 ± 6 h and second measurement at 48 ± 6 h of life (HOL). At each time point echocardiography was performed by a single rater and 3 readings were taken at each time point.

### Method of measuring cardiac output [7]

#### RVO measurement

The parasternal short axis view was used to record flow at a level just distal to the pulmonary valve by Pulsed Doppler. Maximum velocity time integral (VTI) was calculated, by averaging the values of area under the curve, for five consecutive cardiac cycles. The peak to peak intervals of the Doppler velocity time signals was used to calculate the heart rate. By using a frame to frame analysis of the grey-scale parasternal long-axis image, the diameter of the pulmonary valve insertion was measured at end systole. The average diameter was calculated from values of the five cardiac cycles**.**

#### LVO measurement

The apical view was used to image the left ventricular outflow tract so that the full length of the ascending aorta can be incorporated. The range gate of pulsed Doppler was placed distal to the aortic valve and the maximum VTI was calculated by averaging the flow velocity time signal values of five consecutive cardiac cycles. The peak to peak intervals of the Doppler velocity time signals was used to calculate the heart rate. A parasternal long axis view was used to measure the internal diameter of the ascending aorta at the site of flow analysis, at the end of systole, from a frame by frame analysis of the Gray-scale image. An average value of the diameter was calculated from five cardiac cycles.

#### SVC Flow Measurement

The SVC was imaged on its entry to the right atrium, from the subcostal view. SVC diameter measurements were taken and averaged from three to five cardiac cycles. The SVC flow was identified by angling the beam anteriorly until the flow into the right atrium is seen using color Doppler. SVC flow VTI was measured and used to calculate the SVC flow. Ventricular output has been represented as mL/kg/minute. The following formula was used for measuring ventricular output:

Flow = HR x VTI x π (diameter/2)^2^.

Where, Heart Rate (HR), Velocity Time Integral (VTI), π -3.142.

Heart rate was measured by a pulse oximeter attached to right hand of the baby while performing functional echocardiography.

Sample size: All term neonates born intramurally undergoing DCC for 1 min were included in the study.

Bland Altman plot was used to assess intra-observer variability. The limits for intra-observer variability were calculated using mean difference and standard deviation of mean difference. *P*-value < 0.05 has been considered as significant. Intra-class correlation coefficients (ICCs) were also calculated for all measurements. Intra-observer measurement ICCs were calculated by a two-way mixed model with absolute agreement.

### Statistical analysis

Data analysis was performed by using SPSS statistical software version 19:0.Descriptive statistics was used to describe the data. Mean and standard deviation was used for quantitative variables whereas frequency and percentage was used for qualitative variable. Two repeated quantitative data was compared with paired sample t test. Two independent quantitative groups were compared with unpaired t test. Bland Altman plot was used to assess intra and inter observer variability.

### Outcome variables

The primary outcome variables of the study were described as right ventricular output (RVO) (ml/kg/min) at 12 ± 6 and 48 ± 6 h of life, left ventricular output (LVO) (ml/kg/min) at 12 ± 6 and 48 ± 6 h of life, superior vena cava flow (SVC) (ml/kg/min) at 12 ± 6 and 48 ± 6 h of life, heart rate (HR) (bpm) at 12 ± 6 and 48 ± 6 HOL.

## Results

**Fig. 1:**
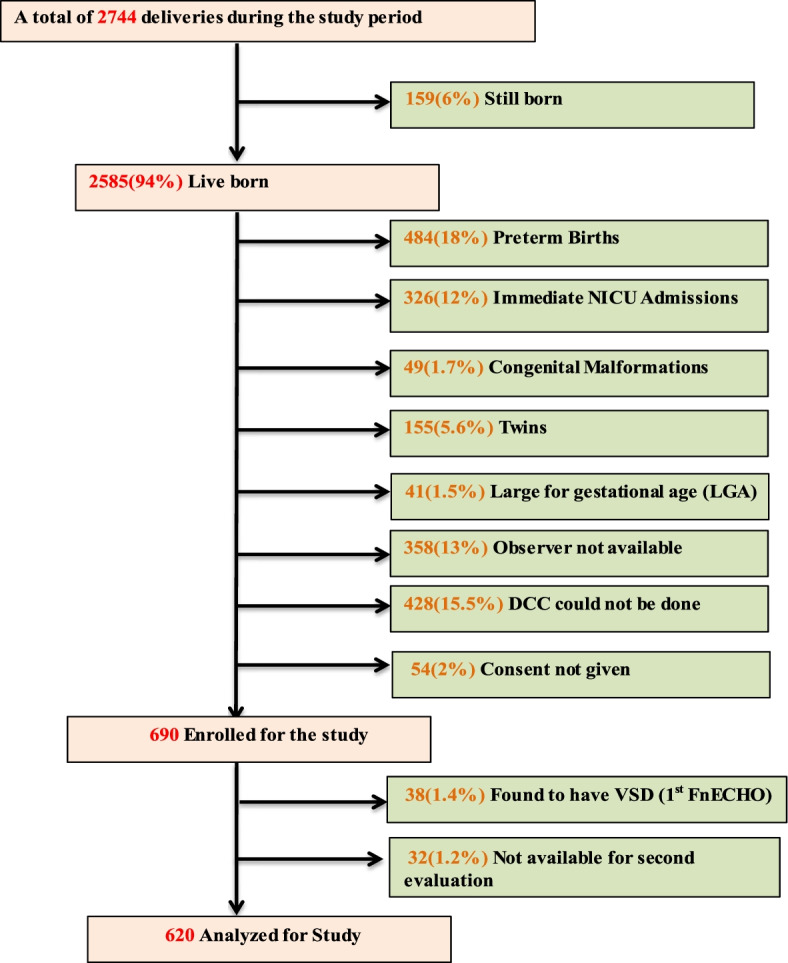
Flow Diagram of patients during the study period

Flow chart of recruitment is depicted in Fig. 1.

Table 1 shows that 239(38.5%) babies were born to primigravida mothers, 381(61.5%) were born to multigravida mothers. Out of total babies enrolled 382(61.6%) were delivered by normal vaginal delivery and 238(38.4%) were born by caesarean delivery due to various reasons. Various maternal morbidities have also been mentioned in Table 1.

**Table 1:** Baseline maternal characteristics (*n* = 620)

**Characteristic**		**n (%)**
Gravida	Primigravida	239 (38.5%)
	Multigravida	381 (61.5%)
Mode of delivery	Normal vaginal delivery	382 (61.6%)
	Caesarean delivery	238 (38.4%)
Pregnancy induced hypertension	Yes	230 (37.1%)
Gestational Diabetes mellitus	Yes	98 (15.8%)
Thyroid Disorder	Yes	74 (11.9%)
Anaemia	Yes	31(5%)
Urinary tract infection/Renal disease	Yes	18 (2.9%)
Heart Disease	Yes	1(0.2%)

Male babies were 298 (48.1%) and 322 (51.9%) babies were female. Male: Female ratio was 1:1.08. Median gestational age was 39(38.1-39.8) weeks. Mean birth weight was 2.9 ± 0.39 kg. Appropriate for gestational age (AGA) babies were 524 (84.5%) of the total whereas 96 (15.5%) babies were small for gestational age (SGA) (neonatal baseline characteristics Table 2).

**Table 2:** Baseline neonatal characteristics (*n* = 620)

Characteristic		n (%)
Gender	Male(M)	298 (48.1%)
	Female(F)	322 (51.9%)
	Ratio(M:F)	1:1.08
Gestational age(weeks)	Median(^5^IQR)	39(38.1–39.8)
Mean Birth weight(Kg)	Kg ± ^4^SD	2.9 (0.39)
^1^SGA	n (%)	96 (15.5%)
^2^AGA	n (%)	524 (84.5%)
APGAR score 1 min	Median(IQR)	8(8–9)
APGAR score 5 min	Median(IQR)	9(9–9)
Temperature during ^3^Fn-Echo (degree Celsius)	Mean ± SD	37 (0.17)
Respiratory rate (per minute)	Mean ± SD	42 (1.76)
SpO2 (Right upper limb) (%)	Mean ± SD	98(0.71)
Immediate skin to skin contact	n (%)	364(58.7%)

^1^*SGA *Small for gestational age, ^2^*AGA* Appropriate for gestational age, ^3^*Fn-Echo* Functional echocardiography, ^4^*SD* Standard deviation, ^5^*IQR* Interquartile range Q1-Q3

Out of 2585 live deliveries, 428 babies were excluded for the need of resuscitation and immediate cord clamping. Average duration of skin to skin contact has not been recorded in this study but routinely 30 min of skin to skin contact is offered as a part of institutional policy. Mean duration of cord clamping was one minute. Cord blood ABG for healthy term neonates is not routinely practiced so pH in umbilical artery could not be calculated.

Mean heart rate (HR), left ventricular output (LVO), right ventricular output (RVO) and Superior vena cava flow (SVC) were measured at two time points, 12 ± 6 h and 48 ± 6 HOL with comparison of 2 time points (Table 3).

**Table 3:** Functional echocardiography measures at 12 ± 6 and 48 ± 6 h of life

**Variable**	**12 ± 6 h of life**	**Lower CI**	**Upper CI**	**48 ± 6 h of life**	**Lower CI**	**Upper CI**	***P* value**
**Heart Rate(bpm)**	126.91 (12.94)	125.89	127.93	128.18 (13.57)	128.13	130.18	0.073
^**1**^**LVO**							
**Diameter(mm)**	0.64 (0.041)	0.64	0.64	0.63 (0.041)	0.63	0.64	< 0.001
^**3**^**VTI(cm)**	9.32 (1.19)	9.23	9.41	9.56 (1.43)	9.44	9.67	< 0.001
^**1**^**LVO(ml/kg/min)**	133.68 (31.15)	131.23	136.14	134.78 (29.84)	132.44	137.13	0.25
^**2**^**RVO**							
**Diameter(mm)**	0.68 (0.05)	0.68	0.69	0.68 (0.051)	0.68	0.68	0.678
^**3**^**VTI(cm)**	12.64 (1.46)	12.53	12.76	12.47 (1.53)	12.35	12.59	0.001
^**2**^**RVO(ml/kg/min)**	209.55 (44.89)	206.02	213.08	205.85 (46.35)	202.21	209.50	0.002
^**4**^**SVC**							
**Diameter(mm)**	0.48 (0.040)	0.48	0.49	0.48 (0.041)	0.48	0.49	0.678
^**3**^**VTI(cm)**	13.14 (2.01)	12.98	13.29	13.31 (1.92)	13.16	13.46	0.003
^**4**^**SVC Flow(ml/kg/min)**	106.85 (26.21)	104.78	108.91	109.29 (25.11)	107.31	111.26	0.001

^1^*LVO* Left ventricular output, ^2^*RVO* Right ventricular output, ^3^*VTI* Velocity time integral, ^4^*SVC* Superior vena cava

**Fig. 2:**
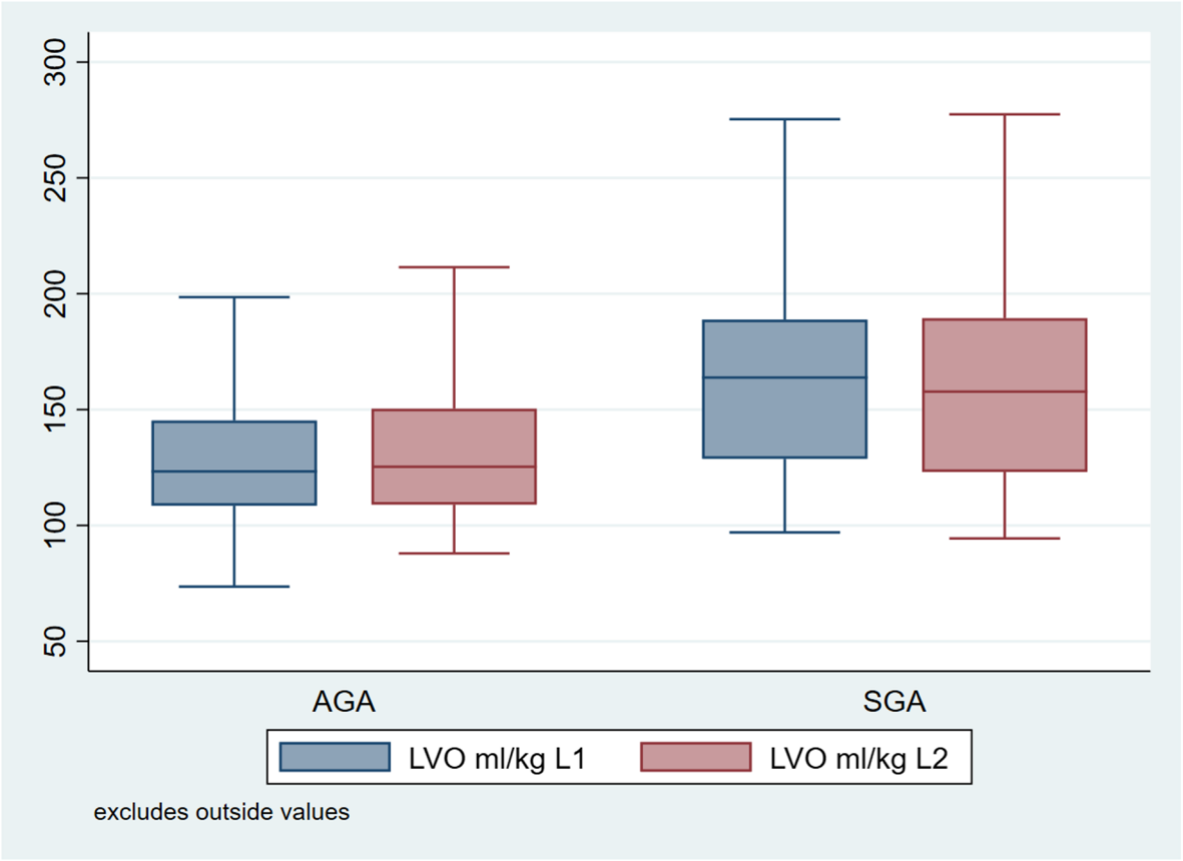
Comparison between LVO of AGA and SGA babies 12 ± 6 h and 48 ± 6 h of life

**Fig. 3:**
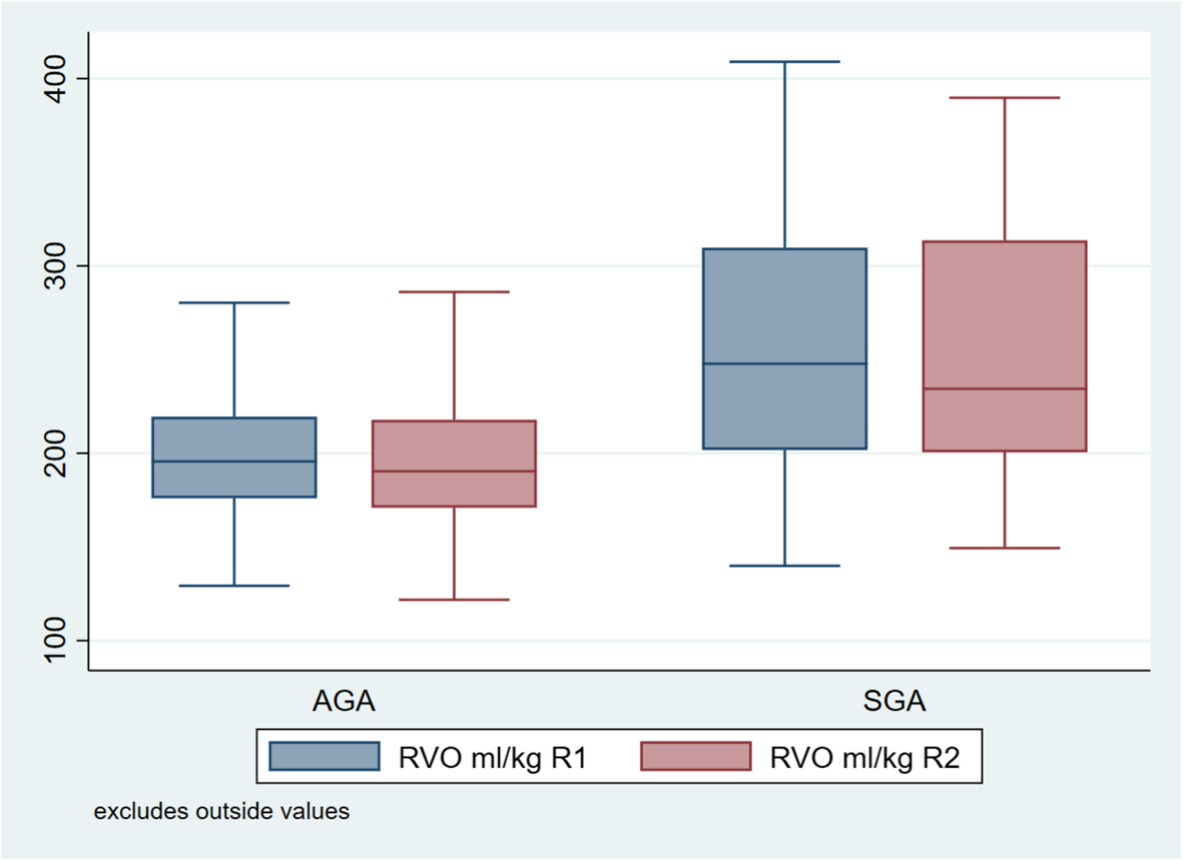
Comparison between RVO of AGA and SGA babies 12 ± 6 h and 48 ± 6 h of life

**Fig. 4:**
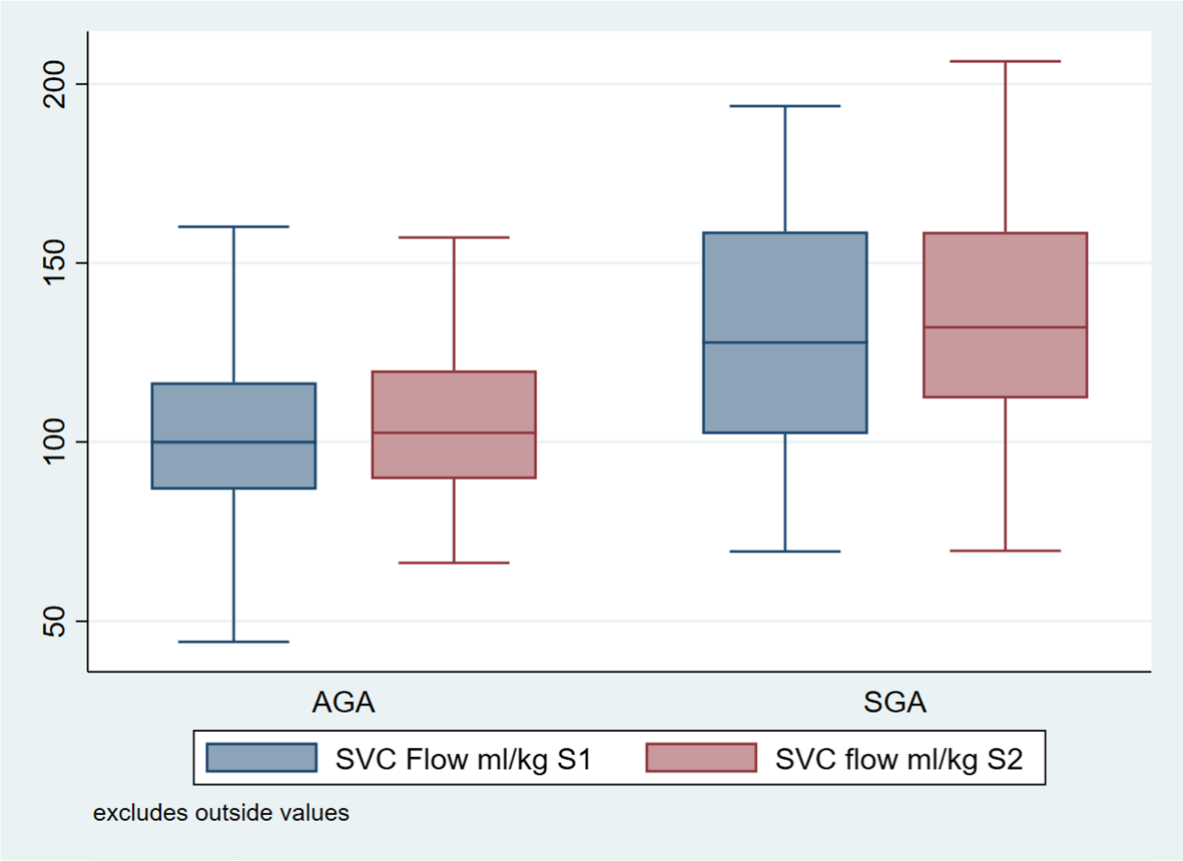
Comparison between SVC flow of AGA and SGA babies 12 ± 6 h and 48 ± 6 h of life

The effect of birth weight for gestation on the outcome parameters in AGA and SGA groups is described in (Table 4, Figs. 2, 3, and 4).

**Table 4:** Comparison between AGA and SGA

**Variable**	**12 ± 6 Hours of life**		**Lower CI**	**Upper CI**	***P*** value
	**AGA**	**SGA**			
**Heart rate(bpm)**	126.01 (12.35)	131.82 (14.89)	-8.53	-2.93	**0.0001**
^**1**^**LVO**					
**Diameter(mm)**	0.64 (0.04)	0.62(0.04)	0.012	0.030	< 0.001
^**3**^**VTI(cm)**	9.37 (1.16)	9.05 (1.32)	0.065	0.582	0.014
^**1**^**LVO(ml/kg/min)**	128.43 (25.53)	162.35(41.88)	-40.436	-27.895	< 0.001
^**2**^**RVO**					
**Diameter(mm)**	0.68 (0.05)	0.67 (0.05)	0.004	0.026	0.0005
^**3**^**VTI(cm)**	12.70(1.4)	12.32(1.7)	0.060	0.697	0.019
^**2**^**RVO(ml/kg/min)**	200.77(34.01)	257.49(63.35)	-66.41	-48.98	< 0.001
^**4**^**SVC**					
**Diameter(mm)**	0.48(0.04)	0.48(0.04)	0.005	0.022	0.305
^**3**^**VTI(cm)**	13.18(1.94)	12.92(2.36)	-0.183	0.696	0.255
^**4**^**SVC Flow(ml/kg/min)**	102.20(21.49)	132.20(34.11)	-35.33	-24.88	< 0.001
**Variable**	**48 ± 6 Hours of life**		**Lower CI**	**Upper CI**	***P* value**
	**AGA**	**SGA**			
**Heart rate(bpm)**	127.67(12.81)	131.01(16.94)	-5.837	-0.151	0.026
^**1**^**LVO**					
**Diameter(mm)**	0.64 (0.04)	0.61(0.04)	0.012	0.030	< 0.001
^**3**^**VTI(cm)**	9.65(1.45)	9.06(1.21)	0.285	0.904	< 0.001
^**1**^**LVO(ml/kg/min)**	130.59(25.55)	157.70(39.74)	-33.655	-21.321	< 0.001
^**2**^**RVO**					
**Diameter(mm)**	0.68 (0.05)	0.67 (0.05)	0.005	0.026	0.0005
^**3**^**VTI(cm)**	12.50(1.5)	12.27(1.7)	-0.097	0.573	0.166
^**2**^**RVO(ml/kg/min)**	197.26(35.15)	252.78(67.56)	-65.805	-47.573	< 0.001
^**4**^**SVC**					
**Diameter(mm)**	0.48(0.04)	0.47(0.03)	0.004	0.021	0.006
^**3**^**VTI(cm)**	13.34(1.84)	13.17(2.27)	-0.235	0.604	0.42
^**4**^**SVC Flow(ml/kg/min)**	104.85(20.91)	133.51(31.64)	-33.94	-23.93	< 0.001

^1^*LVO* Left ventricular output, ^2^*RVO*-Right ventricular output, ^3^*VTI* Velocity time integral, ^4^*SVC* Superior vena cava

SGA babies had a significantly higher heart rate at 12 ± 6 h of life as compared to AGA babies, although this difference in heart rate could not be appreciated at 48 ± 6 h of life. However SGA babies had a higher LVO, RVO and SVC flow than AGA babies at both the time points of observation. However RVO and SVC flow in our study are comparable to RVO and SVC flow of other studies, LVO of SGA babies after delayed cord clamping is found to be significantly lower than LVO seen in other studies.

There was good intra-observer agreement in the measured variables with interclass correlation co-efficient being more than 0.65 in all except for SVC diameter, where it was found to be 0.472 and 0.44 respectively at two time points.

## Discussion

In our study heart rate at two occasions (12 ± 6 h and 48 ± 6 HOL) was 126 (12.94) bpm and 128 (13.57) bpm, left ventricular output (LVO) was found to be a mean (SD) of 133.68 (31.15) and 134.78 (29.84) ml/kg/min respectively, right ventricular output (RVO) was 209.55 (44.89) and 205.85 (46.35) ml/kg/min SVC flow was 106.85 (26.21) and 109.29 (25.11) ml/kg/min respectively.

### Heart rate

In a study by Agata et al. [8] (1991) heart rate was reported to be 145 bpm and 121 bpm at 24 and 96 h of life in term healthy neonates, however the timing of cord clamping has not been mentioned in this study. The heart rate was higher as compared to our study at 24 h of life whereas the heart rate dropped to a lower level by 96 h of life, probably indicating cardiovascular adaption. In another study done by Coksun et al. [9] (2001), which enrolled 45 infants, heart rate was found to be 128 and 126 bpm at 1 and 24 h of life. Van Vonderen JJ et al [10] (2014) studied transitional circulation in term neonates after 30–60 s of cord clamping time and found heart rate at 1 min and 5 min, 10 min of life to be 158,159,156 bpm. Smit et al [11] (2014) in their study reported heart rate at 1 min and 10 min of life to be 161 and 146 bpm respectively. Another study done by katheria et al. [12] (2015) in 20 babies after DCC reported heart rate at 1 min and 5 min of life to be 176 and 168 bpm respectively. Linde et al. [13] (2016) in their study reported heart rate to be 149 at 1 min of life. In another study, by Kee Soo Ha et al. [14] (2018) including 56 infants, heart rate was seen as 136 and 135 bpm at 12 and 48 h of life respectively which are slightly higher than our study. A study by Ashish kc et al. [15] (2019) documented heart rate to be 137 at 10 min of life. Heart rate documented in our study was also comparable to another study (127 vs 120 bpm) done by Stritzke et al. [16] (2019).

It can be deduced that as a result of placental transfusion by DCC, the initial surge of heart rate as reported in previous studies is prevented and heart rate remains stable throughout the transition.

### Left ventricular output (LVO)

The earliest literature about measurement of left ventricular output by Gessner et al. [17] (1965) during transitional circulation in term infants at 2 h of life showed LVO to be 233 ml/kg/min which was higher than the LVO found in our study. In another study by Alverson et al. [18] (1984) involving 14 patients, cardiac output was documented to be 236 ml/kg/min in first week of life. Winberg et al. [19] (1989) in their study involving 16 patients reported cardiac output as 237 and 200 ml/kg/min at 2 and 24 HOL respectively. LVO values in our study were lower than LVO values seen in a study done by Agata et al. [8] (1991) in which cardiac output was found to be 245 ± 56 and 228 ± 44 ml/kg/min at 24 and 96 HOL. Coksun et al. [9] (2001) performed another study and found LVO to be 260 ± 47 at 24 HOL and 249 ± 46 at 72 HOL which was higher than the left ventricular output found in our study. Van Vonderen (2014) [10] in their study involving 24 infants found left ventricular output to be 151 ± 47 ml/kg/min which is same as found in our study. Groves, A. M. et al. [20] (2011) reported cardiac output by cardiac MRI to be 222 ml/kg/min in 28 newborns within first 10 days of life. Popat et al [21] (2012) in their study involving 21 babies at 4 h of life reported left ventricular output to be 193 (148–278) ml/kg/min, which is higher than the value found in our study. In another study by Noori et al. [22] (2012) involving 20 newborns LVO was found as 168 ± 42 ml/kg/min (higher than that found in our study).In a study done by Kee Soo ha et al. [14] (2018) involving 56 patients LVO measured at 12 and 48 HOL was found to be 205 and 189 ml/kg/min (higher than our study). The measures of left ventricular output in our study were however identical to that found by Jain et al. [23] (2018) which involved 15 patients and reported LVO as 140 ± 34 and 143 ± 43 at 7–10 HOL and 22–24 HOL respectively. The results of LVO in our study were similar to a study done by Stritzke et al. [16] (2019) enrolling 26 patients reporting LVO as 141.1 (36.7) at 2–6 HOL and 133.2 (43.3) at 24 HOL.

### Right Ventricular Output (RVO)

The results of RVO in our study were similar to those found in a study done by Groves A. M. et al. [20] (2011) with 28 subjects where RVO in less than 10 days of life was found to be 219 (46.9) ml/kg/min. Popat et al. [21] (2012) in their study with 21 subjects reported RVO to be 216 (122–338) ml/kg/min at four hours of life (almost similar to the value of RVO found in our study). Jain et al. [23] (2018) found RVO to be 218 ± 76 and 250 ± 78 ml/kg/min at 7–10 HOL and 22–24 HOL respectively, approximating the values of RVO found in our study. Stritzke et al. [16] (2019) in their study reported RVO in 26 healthy term infants as 211(60.6) and 223(63.9) ml/kg/min at 2–6 HOL and 24 HOL respectively. These values were almost similar to RVO values found in our study however the timing of cord clamping has not been mentioned in any of the included studies.

### Superior Vena Cava flow (SVC flow)

In a study done by Groves A.M et al. [24] SVC flow was found to be 95 (± 27) ml/kg/min, which is lower than the SVC flow found in our study. Banait et al. [25] (2013) in their study involving 52 term SGA babies reported SVC flow as 126.28 ml/kg/min. In another study done by Kee Soo Ha et al. (2018) [14] enrolling 56 infants with similar baseline characteristics as our study, SVC flow at 12 and 48 HOL was reported as 103 and 99 ml/kg/min approximating the values of our study.

### Why lower heart rate and lower left ventricular output (LVO) after DCC?

The relatively lower values of heart rate and left ventricular output(LVO) found in our study could be deciphered as a result of stabilizing effect of blood transfusion (by delayed cord clamping) on heart rate and cardiac output as seen in other studies.

A study done by Hudson et al. [26] (1990) measuring the cardiac output 12–24 h after blood transfusion in 24 infants found lower cardiac output after blood transfusion (286 ml/kg/min vs 251 ml/kg/min). Nelle et al. [27] (1994) in their study reported a significantly lower heart rate and lower cardiac output after blood transfusion. Another study done by Kanmaz et al [28] (2013) evaluating 35 babies also reported a significantly lower heart rate and lower cardiac output 24 h after blood transfusion.

### Comparison of AGA versus SGA

According to various recent studies it has been found that small for gestational age babies are at a higher risk of neonatal morbidity and are prone to adult onset cardiovascular diseases due to various epigenetic changes in cardiac regulation which hampers a smooth cardiovascular adaptation after birth In our Study SGA babies were 15.5% of the total enrolled babies.

The baseline heart rate of SGA babies found in our study at two time points (12 ± 6 and 48 ± 6 h of life) is comparable to heart rate reported by Guajardo et al.124 (1994) in their study. Left ventricular output (LVO) of SGA babies when analyzed separately was found to be 162.35 and 157.70 ml/kg/min at 12 ± 6 and 48 ± 6 h of life respectively, which is lower than left ventricular output reported by Guajardo et al. [29] (314 and 319 ml/kg/min at day 1 and day 5 respectively). In a study done by Leipälä et al. [30] (2003) LVO was seen as 243 ± 47 ml/kg/min (higher than our study). Banait et al. [25] (2013) reported LVO as 214.61 ml/kg/min in SGA babies after first 7 days of life.Right ventricular output (RVO) in our study at two time points (12 ± 6 and 48 ± 6 h of life) was 257.49 and 252.78 ml/kg/min which is comparable to RVO (255.59 ml/kg/min) reported by Banait et al. [25] (2013) in their study. Regarding SVC flow, we found it to be 132.20 and 133.51 ml/kg/min at two time points (12 ± 6 and 48 ± 6 h of life) as compared to the study done by Banait et al [25] (2013) where it was reported as 126.28 ml/kg/min.

So as compared to AGA babies, SGA babies had a significantly higher baseline heart rate, LVO, RVO and SVC flow. However RVO and SVC flow in our study are comparable to RVO and SVC flow of other studies, LVO of SGA babies after delayed cord clamping is found to be significantly lower than LVO seen in other studies, favoring the cardio-stabilizing effect of DCC.

There was a moderate to good (ICC 0.5–0.9) intra-observer agreement for all the variables except for SVC diameter and SVC flow. The large intra-observer variation seen in measurement of superior vena cava diameter found in our study is in coherence with the other studies [24, 31, 32] reporting superior vena cava flow measurements. The reasons for this variation could be difficulty in capturing the view of superior vena cava due to interference of ultrasound image acquisition from neonate’s inflated lungs. Moreover, due to lack of muscle within the venous vessel wall and compression of the superior vena cava by the aorta, the cross-sectional area might be D shaped and not truly circular as opposed to aortic valve or pulmonary valve annulus.

The antecedent studies evaluating the cardiac functions of term neonates during transitional circulation have not considered the timing of cord clamping (early or delayed), which alter the hemodynamics of the neonate significantly. As DCC has proven benefits for term and preterm infants, subjecting few infants to ICC for the study purpose and depriving them of the benefits of DCC for would have been unethical that’s why no comparison of the two methods of cord clamping was done. This is the first study of its kind to ascertain the changes in cardiac hemodynamics of term babies undergoing DCC. With 620 babies being analyzed this is probably the largest study estimating the cardiac outputs in babies who have undergone DCC.

### Strengths of the study

- Large sample size has been analyzed
- Inter-observer as well as intra-observer variability in the various parameters has been measured and documented.

### Limitations of the study

- It is an observational study involving only infants who have undergone delayed cord clamping, as a randomized controlled trial of early versus delayed cord clamping as it would have been unethical to not offer DCC.
- The pattern and direction of PDA which might have influenced the right and left ventricular outputs during the transitional circulation has not been documented.
- High intra-observer and inter-observer variability seen in measurement of SVC flow.

### Way Forward

Further study evaluating the quantitative assessment of LV function using fraction shortening (FS), ejection fraction (EF), Doppler pattern of LV filling (E and A waves at mitral valve), and tissue Doppler imaging (TDI) in infants undergoing DCC needs to be undertaken. Future studies may consider integration of information like respiratory rate and behavior of the infant (eg, crying), which influence VTI values obtained for various measurement. Placental transfusion by way of DCC in term infants has beneficial effects in terms of cardiovascular stabilization, similar to packed red blood cell transfusion. Given the adaption of DCC as a standard of care globally, the normative data of heart rate and cardiac output of term, stable babies requires further evaluation.

## Conclusion

During the study period, 620 neonates’ haemodynamic parameters were recorded after delayed cord clamping. Heart rate and LVO were lower than other studies, reflecting cardiovascular stability as a result of DCC. Lack of difference in the heart rate and cardiac output between 12 ± 6 and 48 ± 6 h of life implied less fluctuations and a better stability in the cardiovascular status right after birth. Small for gestational age (SGA) babies had a higher baseline heart rate and cardiac output as compared to appropriate for gestational age (AGA) babies, warranting a new nomogram for heart rate and cardiac output for SGA babies. In, conclusion delayed cord clamping by the virtue of placental transfusion reduces fluctuation in the hemodynamic parameters (heart rate, cardiac output) at the two time points of observation. Hence, while evaluating the cardiac output of an infant, timing of cord clamping must be taken into account and a new normative data of heart rate and cardiac output for neonates after DCC needs to be established.

## Data Availability

The datasets used and/or analyzed during the current study are available from the corresponding author on reasonable request.
